# Retraction Note to: An experimental study on stress-shielding effects of locked compression plates in fixing intact dog femur

**DOI:** 10.1186/s13018-022-03307-x

**Published:** 2022-09-07

**Authors:** Xinwen Zhao, Wensen Jing, Zhe Yun, Xun Tong, Zhao Li, Jiajia Yu, Yaohui Zhang, Yabin Zhang, Zhixue Wang, Yanhua Wen, Heping Cai, Jun Wang, Baoan Ma, Haien Zhao

**Affiliations:** 1 Department of Joint Surgery, Yuncheng Hospital, The Eighth Clinical Medical University, Yuncheng, 441000 Shaanxi Province China; 2 Department of Orthopedics, Honghui Hospital, Xi’an Jiaotong University, 555 Youyi East Road, Beilin District, Xi’an, 710054 Shaanxi Province China; 3 Department of Orthopedics, 941st Hospital of PLAJLSF, Xining, 810000 Qinghai Province China; 4 Department of Traditional Chinese Medicine Rehabilitation and Physiotherapy, Xuzhou Army 71st Group Army Hospital, Xuzhou, 221000 Jiangsu Province China; 5 Institution of Orthopedics, Tangdu Hospital, Air Force Medical University, Xi’an, 710038 Shaanxi Province China; 6 School of Materials Science and Engineering, Xi’an Jiaotong University, Xi’an, 710061 Shaanxi Province China; 7 Center of Orthopedic Laboratory, Xijing Hospital, Air Force Medical University, Xi’an, 710038 Shaanxi Province China

## Retraction Note to: Journal of Orthopaedic Surgery and Research 16:97 (2021)10.1186/s13018-021-02238-3

The Editor-in-Chief has retracted this article. Contrary to the statement in the article, this study was not approved by the Institutional Animal Care and Use Committee of Tangdu Hospital, The Air Force Medical University. The corresponding author has informed the Journal that at the time the study was performed there was no procedure for obtaining ethical approval. The corresponding author Baoan Ma has stated on behalf of all coauthors that they agree with this retraction.

